# Naphthalimide-Containing BP100 Leads to Higher Model Membranes Interactions and Antimicrobial Activity

**DOI:** 10.3390/biom11040542

**Published:** 2021-04-08

**Authors:** Gustavo Penteado Battesini Carretero, Greice Kelle Viegas Saraiva, Magali Aparecida Rodrigues, Sumika Kiyota, Marcelo Porto Bemquerer, Hernan Chaimovich, Iolanda Midea Cuccovia

**Affiliations:** 1 Departamento de Bioquímica, Instituto de Química, Universidade de São Paulo, São Paulo, SP 05508000, Brazil; gustavo.carretero@usp.br (G.P.B.C.); greice.kelle@gmail.com (G.K.V.S.); magarodrig@gmail.com (M.A.R.); 2 Laboratório de Bioquímica de Proteínas e Peptídeos, Instituto Biológico, São Paulo, SP 04014002, Brazil; sumika.kiyota@sp.gov.br; 3 Embrapa Recursos Genéticos e Biotecnologia, Parque Estação Biológica, Empresa Brasileira de Pesquisa Agropecuária, Brasília, DF 70770900, Brazil; marcelo.bemquerer@embrapa.br

**Keywords:** antimicrobial peptide, BP100, model membranes, spectroscopy, calorimetry, biological activity, naphthalimide

## Abstract

In a large variety of organisms, antimicrobial peptides (AMPs) are primary defenses against pathogens. BP100 (KKLFKKILKYL-NH_2_), a short, synthetic, cationic AMP, is active against bacteria and displays low toxicity towards eukaryotic cells. BP100 acquires a α-helical conformation upon interaction with membranes and increases membrane permeability. Despite the volume of information available, the action mechanism of BP100, the selectivity of its biological effects, and possible applications are far from consensual. Our group synthesized a fluorescent BP100 analogue containing naphthalimide linked to its N-terminal end, NAPHT-BP100 (Naphthalimide-AAKKLFKKILKYL-NH_2_). The fluorescence properties of naphthalimides, especially their spectral sensitivity to microenvironment changes, are well established, and their biological activities against transformed cells and bacteria are known. Naphthalimide derived compounds are known to interact with DNA disturbing related processes as replication and transcription, and used as anticancer agents due to this property. A wide variety of techniques were used to demonstrate that NAPHT-BP100 bound to and permeabilized zwitterionic POPC and negatively charged POPC:POPG liposomes and, upon interaction, acquired a α-helical structure. Membrane surface high peptide/lipid ratios triggered complete permeabilization of the liposomes in a detergent-like manner. Membrane disruption was driven by charge neutralization, lipid aggregation, and bilayer destabilization. NAPHT-BP100 also interacted with double-stranded DNA, indicating that this peptide could also affect other cellular processes besides causing membrane destabilization. NAPHT-BP100 showed increased antibacterial and hemolytic activities, compared to BP100, and may constitute an efficient antimicrobial agent for dermatological use. By conjugating BP100 and naphthalimide DNA binding properties, NAPHT-BP100 bound to a large extent to the bacterial membrane and could more efficiently destabilize it. We also speculate that peptide could enter the bacteria cell and interact with its DNA in the cytoplasm.

## 1. Introduction

Antimicrobial peptides (AMPs) can destroy or inhibit the growth of bacteria, fungi, and viruses [1]. AMPs are ubiquitous components of the innate immune system and act as endogenous antibiotics [2,3,4,5]. AMPs are usually positively charged and display a hydrophobicity index and hydrophobic moment, compatible with interactions with the bacterial membrane [6,7]. Membrane interaction usually triggers peptide folding; a large variety of structures can be found in natural and synthetic AMPs such as α-helix, β-sheet, and bend or turn-like folds [1,2,3,4,5,6,7]. The antibacterial activity of AMPs arises from electrostatic interactions with bacterial membranes, rich in negatively charged components such as phosphate, lipopolysaccharides from Gram-negative bacteria, or lipoteichoic acids in Gram-positive bacteria. As the negative charge density of mammalian cell membranes is lower than that of bacteria, the electrostatic component is the main element of selectivity towards bacteria in cationic peptides’ action [3,4,5,6].

Peptide flip and hydrophobic residue exposure to the membrane interior may follow electrostatic binding [8]. AMPs, in addition to the high density of positively charged side chains, contain membranotropic aromatic tryptophan, tyrosine, and phenylalanine residues [2,4,5,9]. After interaction with the membrane, the AMPs hydrophobic/hydrophilic topological distribution acquires a secondary structure that provides the peptide with a spatial amphipathic character, favoring the interaction with the membrane interface [4,7]. Dehydration of the hydrophobic moieties, and not just the electrostatic components, can determine the bonding selectivity of AMPs to bacterial membranes [10].

**Figure 1:**
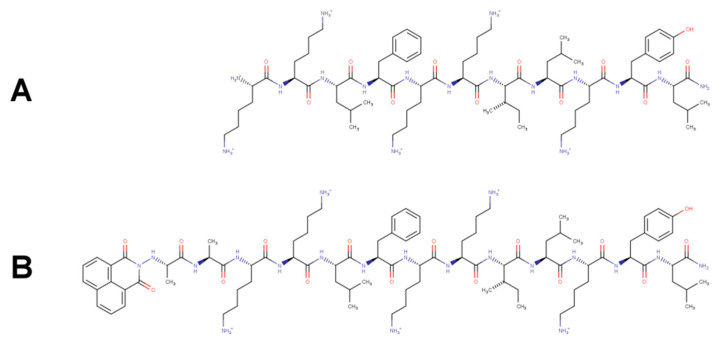
Structures of BP100 (KKLFKKILKYL-NH_2_) (**A**) and Naphthalimide-AA-BP100 (1,8-Naphthalimide-AAKKLFKKILKYL-NH_2_)(NAPHT-BP100) (**B**).

Badosa and coworkers designed a series of AMP’s to identify antimicrobial structure/potency relationships [11]. BP100 (KKLFKKILKYL-NH_2_) (Figure 1A) combines the sequence of melittin and cecropin A, acts by inhibiting the bacterial growth, exhibits low toxicity, high therapeutic index, and low sensitivity to degradation [12]. Atomic force microscopy showed that BP100 destroys the bacterial outer envelope at a minimum inhibitory concentration (MIC) of 3 μM [13]. The extent of damage is related to peptide binding and neutralization of the cell membrane’s surface charge. Circular dichroism (CD) and in silico analysis showed that the membrane-bound form of BP100 had an α-helix content of 61% [13,14,15,16,17]. The acquisition of α-helical secondary structure results in an amphipathic structure, ideal for peptide/negatively charged lipid bilayer interaction. Oriented circular dichroism (OCD) and solid-state nuclear magnetic resonance (SS-NMR) of BP100 labeled with ^19^F showed that the helix is positioned on the membrane with its long axis parallel to the membrane surface [16]. After an initial electrostatic driven approach, BP100 acquires helical conformation and accommodates at the interface by flipping along its helix longer axis and inserting the hydrophobic face down to the membrane hydrophobic acyl chain depth. Two other relevant phenomena regarding binding and flipping processes were also accessed: negative lipid clustering and peptide dehydration [8]. Microorganisms, or transformed cell disruption by BP100, and several BP100 analogs, and the effects resulting from groups linked to the parental peptide [17,18], were analyzed [18].

Here, we present biophysical and biological studies of a naphthalimide-conjugated BP10 peptide (1,8-Naphthalimide-AAKKLFKKILKYL-NH_2_, NAPHT-BP100) (Figure 1). Naphthalimide (1H-benzo [de] isoquinoline-1,3-(2H)-dione) and related compounds bind to DNA and exert antitumor, anti-inflammatory, antidepressant, antiprotozoal, and antiviral activities [19,20,21,22,23]. Advances in the synthesis of naphthalimide analogs have made it possible to explore derivatives such as mono-naphthalimides, di-naphthalimides, and naphthalimides conjugated with other compounds that exhibit different degrees of antibacterial activity depending on the attributed modifications [24,25,26].

However, the conjugating antimicrobial potential of an AMP and a DNA binding motif such as naphthalimide [27,28,29] has been overlooked. In this work, by taking advantaged of biophysical and biological properties of naphthalimide, we aim to expand the understanding of NAPHT-BP100 and BP100 action mechanism, and to obtain an improved antibacterial agent combining both membrane disruption and DNA binding capabilities.

Our investigation focused on NAPHT-BP100 secondary structure, membrane positioning, and lipid bilayer composition effect on binding extent and thermodynamics. We also examined the peptide effect over membrane properties as lipid packing, liposome surface charge, and peptide-induced liposome size changes. Binding data were correlated with NAPHT-BP100-induced vesicle permeabilization, allowing more accurate discussion of NAPHT-BP100 activity in terms of bound-peptide/lipid ratios. The peptide–DNA interaction was also investigated. Biological activity was examined by determining the minimum inhibitory peptide concentration against Gram-negative and Gram-positive bacterial species. Hemolytic activity against human red blood cells was measured as mean of evaluating peptide toxicity.

## 2. Materials and Methods

### 2.1. Reagents

5(6)-Carboxyfluorescein (CF) (Sigma-Aldrich, St. Louis, MO, USA), was purified, and the sodium salt was prepared and quantified as described previously [15]. 1-Palmitoyl-2-oleoyl-*sn*-glycero-3-phosphocholine (POPC), 1,2-dipalmitoyl-*sn*-glycero-3-phosphocholine (DPPC), 1-palmitoyl-2-oleoyl-*sn*-glycero-3-[phospho-rac-(1-glycerol)], sodium salt (POPG), and 1,2-dipalmitoyl-*sn*-glycero-3-[phospho-rac-(1glycerol)], sodium salt (DPPG) (Avanti Polar Lipids, Alabaster, AL, USA) were used as received. Double-stranded DNA pET-28a(+) vector (ds-DNA) was obtained from Sigma-Aldrich (St. Louis, MO, USA).

### 2.2. Peptide Synthesis

Peptides were synthesized by the solid phase [30], using the “Rink Amide” resin (Peptides International, Louisville, KY, USA) for amidated peptides. Fmoc deprotection reactions were carried out with a 20% solution of 4-methylpiperidine in N,N-dimethylformamide (DMF) for 20–30 min (in two steps of 10–15 min). Coupling reactions were conducted with 1,3-diisopropylcarbodiimide (DIC) and ethyl-2-cyano-2-(hydroxyimino) acetate (Oxyma^®^) or [benzotriazol tetrafluoroborate-1-yloxy(dimethylamino)methylidene]-dimethylazanium (TBTU) and N,N’-diisopropylethylamine (DIEA) in DMF for 60–90 min. Deprotection and coupling steps were monitored by the ninhydrin reaction [30,31]. After each deprotection and coupling step, the resin was washed three times with methanol (or 2-propanol) and DMF, consecutively.

Modifications with naphthalic anhydride were carried out as the final stage of solid-phase synthesis [32,33]. Modifications of the peptides with 1,8-naphthalic anhydride were carried out in DMF at 60 °C for 24 h under orbital agitation, with a molar excess of four to eight times of the anhydride relative to the amino terminal group. A recoupling was carried out with the addition of DIC in equimolar amounts relative to naphthalic anhydride at 60 °C for 24 h. After the synthesis was completed, the final deprotection and cleavage reactions were carried out in a solution of trifluoroacetic acid in the presence of nucleophiles (e.g., triisopropylsilane, 1,2-ethanedithiol, and thioanisole) as carbocation scavengers, for 120 min, at room temperature. After precipitation of the crude material with diisopropyl ether and four to six washes with the same solvent, the peptide was collected by filtration in a porous plate funnel, extracted with water or aqueous acetonitrile solution and lyophilized.

Peptides were purified by reversed-phase chromatography on a semipreparative column with an octadecylsilane matrix (C18, 250 mm × 22 mm, 10 µm, 300 Å) from Grace-Vydac (Columbia, MD, USA) at a flow rate of 9.0 mL/min at room temperature. The purity analysis of the synthetic peptides was conducted by reversed-phase chromatography using an analytical column octadecylsilane (C18, 250 mm × 4.6 mm, 5 µm) Grace-Vydac, using a flow rate of 1.0 mL/min at room temperature (Figures SM-1 and SM-2). The elution, both for the analytical method and for the preparation, was carried out with an increasing gradient of acetonitrile in water, in the presence of trifluoroacetic acid forming ionic pairs with peptide’s cationic groups [34] with detection at 220 nm. The identity of the products was verified by mass spectrometry in the MALDI-TOF mode (Autoflex Speed, Bilerica, MA, USA) (Figures SM-3 and SM-4).

### 2.3. Peptide and ds-DNA Solutions

Peptide solutions were prepared by weighing the dry powder and solubilizing it in autoclaved deionized water. Peptide concentrations were measured using an N-1000 Nanodrop spectrophotometer (Thermo Fisher Scientific Wilmington, DE, USA), considering the tyrosine residue absorbance at 275 nm (ε_275 nm_ = 1400 M^−1^ × cm^−1^) for BP100, and naphthalimide moiety absorbance at 342 nm for NAPHT-BP100 (ε_342 nm_ = 12900 M^−1^ × cm^−1^). The NAPHT-BP100 UV absorption spectrum was recorded in a Varian Cary 50 UV/Vis Spectrophotometer (Agilent Technologies, Santa Clara, CA, USA) from 220 to 500 nm.

The DNA concentration was determined spectrophotometrically using an N-1000 Nanodrop spectrophotometer at 260 nm (ε_260_ = 0.020 (μg/mL)^−1^ × cm^−1^), and the ratio between the absorbance at 260 and 280 nm was used to attest the sample purity. DNA concentration in ng/µL was converted in the number of base pair per volume (bp/L) for stoichiometric analysis of the peptide–DNA interaction.

### 2.4. Model Membrane Preparation

Lipid stock solutions were prepared in chloroform and quantified by measuring phosphate concentration [35]. Lipid films were prepared by mixing an adequate volume of each lipid stock solution, followed by solvent evaporation under a stream of argon, and complete drying under vacuum for at least two hours.

Lipid films were suspended in aqueous Tris-HCl 10 mM, pH 7.4, buffer solution yielding multilamellar vesicles (MLVs). Large unilamellar vesicles (LUVs) with a hydrodynamic diameter of 100 nm were obtained by extrusion of MLV through polycarbonate membranes (Millipore, MA, USA) in a LiposoFast syringe-driven extruder (AVESTIN, Ottawa, Canada).

For the CF leakage assay, LUV were prepared in 10 mM Tris-HCl buffer, pH 7.4 containing CF 50 mM. Bulk solution-free CF was separated from the LUV by size-exclusion chromatography using a prepacked Sephadex G-25 filter column (GE Healthcare, Buckinghamshire, UK) equilibrated with 10 mM Tris-HCl buffer, pH 7.4, with 300 mM NaCl. The collected lipid suspension was quantified by measuring phosphate [35].

### 2.5. Fluorescence

Steady-state fluorescence spectra of NAPHT-BP100 in 10 mM Tris-HCl buffer, pH 7.4, containing or not containing 300 mM NaCl, were obtained using a Hitachi F7000 spectrofluorometer (Hitachi, Tokyo, Japan) at 25, 45, and 65 °C. Naphthalimide moiety fluorescence emission was recorded from 345 to 550 nm, at a rate of 240 nm/min, exciting the sample at 342 nm. The initial peptide concentration was 5, 10, 20, or 40 µM and lipid concentration was varied from 0 to 2.8 mM. To study the peptide–DNA interaction, initial peptide concentration was 5 µM and ds-DNA concentration was varied from 0 to 15 ng/µL.

Spectra of 10 mM Tris-HCl buffer, pH 7.4 and of LUV, or ds-DNA, in buffer without the peptide were obtained and subtracted from the spectra of NAPHT-BP100 for correction. In addition, spectra were also corrected by peptide concentration as lipid addition proceeded.

From both fluorescence and CD spectroscopic data, the fraction of peptide bound to the LUV at any given lipid concentration was calculated by normalizing the fluorescence emission intensity or [θ]_222_ at increasing lipid concentrations, as described in Equation (1).

Fraction Bound_[L]_ = (I_[L]_ − I_0_) / (I_Max_ − I_0_) (1)

where I_0_, I_[L]_, and I_Max_ are the steady-state Tyr fluorescence emission intensities at 387 nm, or [θ]_222_, in solution without lipid (I_0_), at given lipid concentration (I_[L]_) and at the maximum lipid concentration (I_Max_) in the experiment, respectively.

The ratios between bound peptide and lipid (r), and the concentration of free peptide ([Pep]Free) at any given point of the titration were then calculated and plotted to obtain the binding isotherms of the peptide–membrane interaction. Apparent binding constants (K_App_, M^−1^) were calculated considering a simple partition model and the initial slope of the isotherms by using Equation (2) [36,37,38].

r = K_App_ × [Pep]_Free_ (2)

### 2.6. Circular Dichroism

Circular dichroism spectra of NAPHT-BP100 in 10 mM Tris-HCl buffer, pH 7.4 were obtained using a Jasco J-720 spectropolarimeter (Jasco, Easton, MD, USA) at room temperature. Samples were placed in a 1.00 mm optical length quartz cells and spectra were scanned from 190 to 260 nm, at a rate of 50 nm/min, with bandwidth of 2 nm, step resolution of 0.5 nm, response time of 2 s, and the final spectrum was the average of six scans. Initial peptide concentration was 20 µM, and lipid concentration was varied from 0 to 1.6 mM. Spectra of the buffer and the vesicles in buffer were obtained under the same conditions and subtracted from the CD spectra of the peptide. Finally, the ellipticity intensities (θ, mdeg) were normalized to molar ellipticity ([θ], deg.cm^2^.dmol^−1^) using Equation (3) to eliminate the spectral dependence on optical length, peptide concentration and number of residues.

[θ] = θ / (10 × C × l × N)(3)

where C is peptide concentration in mol/L, N is the number of residues, and l is the cell optical length in cm.

### 2.7. Dynamic Light Scattering (DLS)

LUV hydrodynamic diameter and size distribution, and electrophoretic mobility were measured in a Zetasizer Nano apparatus equipped with a 633 nm laser (Malvern, Worcestershire, UK). The LUV surface zeta potential was calculated from the electrophoretic mobility using Henry’s equations (Equations (4) and (5)).

UE = 2 × Ɛ × Z × f(ka) / 3ƞ(4)

ζ = ƞ × UE / Ɛ(5)

where ζ is the zeta potential, UE is the electrophoretic mobility, Ɛ the dielectric constant of water, f(ka) is Henry’s function, and ƞ is the viscosity of the medium.

LUV composed of POPC:POPG (50:50, mol:mol) were prepared in 10 mM Tris-HF buffer, pH 7.4. Lipid concentration remained fixed at 50 µM throughout the experiment and peptide concentration was varied from 0 to 32 µM.

### 2.8. Isothermal Titration Calorimetry (ITC)

ITC measurements were performed at 25 °C in a MicroCal VP-ITC isothermal titration calorimeter (MicroCal, Northampton, MA, USA) by loading a 3–6 mM LUV suspension in the syringe and titrating a 40 µM peptide solution in 10 mM Tris-HCl buffer, pH 7.4, placed in the cell (V_Cell_ = 1.54 mL). Titration consisted of 25 sequential injections of 10 µL of the lipid suspension into the cell every five min. Samples were previously degassed and a reference cell filled with demineralized water.

### 2.9. Differential Scanning Calorimetry

The phase transition temperature (Tm), cooperativity (ΔT_1/2_), and enthalpy (ΔH) of the gel to liquid crystalline phase transition of DPPC:DPPG (70:30, molar ratio) MLV were determined on a MicroCal VP-DSC differential scanning microcalorimeter (MicroCal, Northampton, MA, USA). Samples were prepared by suspending the lipid film in previously degassed 10 mM Tris-HCl buffer, pH 7.4 solution of 0, 6, 12, or 25 µM NAPHT-BP100, to a final lipid concentration of 1.0 mM. Measurements were performed under a constant external pressure of 35 psi in order to avoid bubble formation. Samples were heated at a constant scan rate of 12 °C/h and the temperature was scanned from 15 to 60 °C. Tris-HCl 10 mM buffer, pH 7.4, was used as a reference. The data were analyzed using the Origin 8.5 program.

### 2.10. Model Membrane Permeabilization

CF leakage assays were performed in a black-bottom opaque 96-well plate in which a two-fold serial dilution of the peptide in 10 mM Tris-HCl buffer, pH 7.4 with 300 mM NaCl was mixed with the same volume of the suspension of LUV containing CF with a lipid concentration of 40 µM. Final peptide concentration ranged from 16 to 0.125 µM and the final lipid concentration was 20 µM. Vesicle permeabilization by the peptide triggered CF leakage that, once diluted, increased its fluorescence emission at 520 nm, when exciting the sample at 490 nm. Fluorescence emission was registered for 60 min, at 37 °C, in a Bio-Tek Synergy HT Microplate Reader (Bio-Tek, Winooski, VT, USA). Emission intensity of total permeabilization of the LUV was measured after addition of 1.5 µL of a solution of polidocanol 10% (*v*/*v*) to each well and used as positive control; buffer solution was used as the negative control and to ensure that no spontaneous leakage occurred to a significant extent. The percentage of CF leakage in each well was calculated using Equation (6).

CF Leakage (%) = 100 × (F_P_ − F_0_) / (F_T_ − F_0_) (6)

where F_P_ is the fluorescence intensity of the well containing the peptide after 30 min, and F_0_ and F_T_ are the fluorescence intensities of the negative (before peptide addition) and positive (after polidocanol addition and 100% permeabilization) control, respectively.

### 2.11. Minimum Inhibitory Concentration Assay

The assays for determining the minimum peptide concentration necessary to inhibit bacterial growth were carried out according to Wiegand et al. [39]. *Escherichia coli* (ATCC 25922), *Staphylococcus aureus* (ATCC 25923), and *Bacillus subtilis* (PY79) bacterial species were tested for the assays, carried out in triplicate.

Initially, 10 mL of Müller–Hinton liquid broth (MHB) medium was inoculated with a small number of bacteria from a single colony deposited on a solid agar plate. The bacteria were then incubated overnight at 37 °C and 250 rpm shaking. The cell suspension was then diluted 50 times in MHB medium and incubated again at 37 °C. Upon reaching an optical density in 600 nm of approximately 0.4, the cell suspension was again diluted 250 times in MHB medium, resulting in a bacterial suspension of 10^6^ CFU/mL. In parallel, in a 96-well polypropylene U bottom plate, a two-fold serial dilution of the peptide in MHB medium was prepared with two times the desired final peptide concentration, ranging between 32 and 0.05 μM and a volume of 50 μL. Next, 50 μL of the cell suspension were added to the peptide solution, resulting in a 5 × 10^5^ CFU/mL bacterial suspension. Bacteria suspension added to the 96-well plate had its final concentration checked by performing a 1000-fold dilution, from approximately 10^6^ to 10^3^ CFU/mL, and plating 10 μL spots in the solid LB-agar plate that was then incubated at 37 °C, for 18 h. The number of colonies at each spot was counted and normalized to give the final bacteria concentration used in the assay. MHB inoculated with 50 μL of the bacterial suspension was used as a positive control and sterile MHB as a negative control. After 18 h, bacterial growth was visually checked to access the MIC.

### 2.12. Hemolytic Activity

The hemolytic activity of BP100 and NAPHT-BP100 was evaluated following Mojsoska et al. [40]. Five milliliters of human blood obtained from healthy volunteers were mixed with 40 mL of sterile phosphate buffered saline solution (140 mM NaCl, 10 mM phosphate, pH 7.4, PBS) and centrifuged for 10 min at 1500 rpm, equivalent to 500× *g*. The supernatant was removed and the cells were washed (×3).

Next, 9.7 mL of PBS was added, resulting in a 3% cell volume suspension. A 96-well polypropylene plate (Corning, NY, USA) was previously prepared with serial dilutions of the peptide in PBS at concentrations between 128 and 2 μM and a final volume of 50 μL. For the assay, 50 μL of the 3% red blood cell (RBC) suspension was added to each well of the plate containing the peptide dilutions, yielding a final suspension of 1.5% of RBC. Sterile PBS was used as a negative control, and as a positive control, to achieve 100% hemolysis, 0.1% (*v*/*v*) Triton X-100 was added. The plate was incubated under shaking for 3 h at 37 °C and then centrifuged at 1200 rpm (400*g*) for 10 min. The supernatant containing released solution-free hemoglobin was transferred to a flat-bottomed 96-well polystyrene plate (Greiner Bio-One, Kremsmünster, Austria). The absorbance at 414 nm was then measured on a Synergy HT plate reader (Bio-Tek, Winooski, VT, USA). The percentage of hemolysis was calculated based on the absorbance measured using Equation (7):

Hemolysis (%) = 100 × (A_PEP_ − A_NEG_) / (A_POS_ − A_NEG_) (7)

where A_PEP_, A_NEG_, and A_POS_ refer to the sample’s absorbance with the peptide, and the positive and negative controls, respectively.

## 3. Results

### 3.1. NAPHT-BP100 Membrane Interaction—Binding Extent and Secondary Structure

#### 3.1.1. UV-Absorption and Fluorescence—NAPHT-BP100 Membrane and ds-DNA Binding

**Figure 2:**
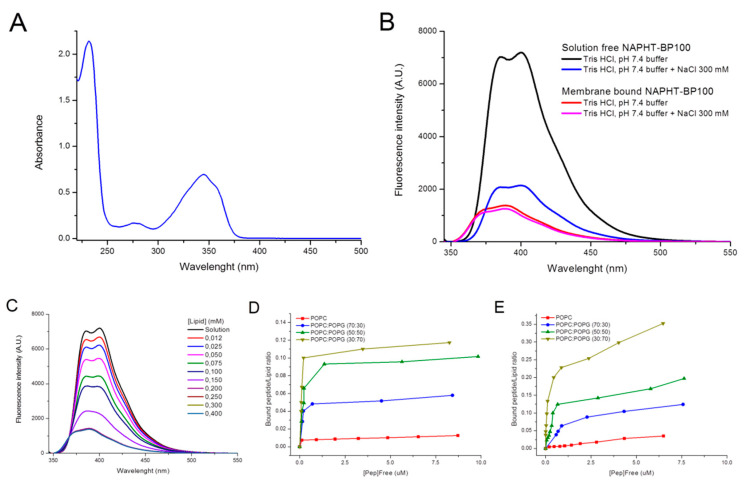
Analysis of NAPHT-BP100 UV absorption, fluorescence, and lipid binding. (**A**) NAPHT-BP100 UV absorption spectrum. Peptide concentration 55 µM. (**B**) Fluorescence spectra of NAPHT-BP100 in solution and bound to POPC:POPG (50:50, molar ratio) large unilamellar vesicle (LUV) in 10 mM Tris-HCl buffer, pH 7.4 with or without 300 mM NaCl. (**C**) Fluorescence spectra of NAPHT-BP100 in solution and with POPC:POPG (50:50) LUV in 10 mM Tris-HCl buffer, pH 7.4. (**D**) Binding isotherms of NAPHT-BP100 to LUV in 10 mM Tris-HCl buffer, pH 7.4. (**E**) Binding isotherms of NAPHT-BP100 to LUV 10 mM Tris-HCl buffer, pH 7.4 with 300 mM NaCl. The peptide concentration was 20 µM and temperature 25 °C. λ _Exc_ = 342 nm.

The NAPHT-BP100 UV absorption spectrum shows three characteristic peaks at 343, 275, and 230 nm corresponding to the absorption from the naphthalimide moiety, the tyrosine side chain, and the peptide bond, respectively (Figure 2A). From this result, the maximum absorption wavelength of the naphthalimide moiety was determined and used as the excitation wavelength to study peptide fluorescence properties.

The emission spectrum of NAPHT-BP100 presented peaks at 385 and 400 nm, and the fluorescence emission intensity was lower at high salt concentrations (Figure 2B). The fluorophore was likely less hydrated in 0.3 M NaCl, causing a decrease in fluorescence emission intensity but no peak displacement [41]. As the temperature increased from 25 to 65 °C, the fluorescence emission of NAPHT-BP100 in solution decreased slightly with no changes in peak positioning or spectral shape (Figure S5A).

Upon POPC: POPG (50:50, molar ratio) LUV addition, NAPHT-BP100 fluorescence emission intensity decreased, and both emission peaks were blue-shifted by 10 nm (Figure 2B,C). Blue shifts and decrease in emission intensity decrease are associated with non-substituted 1,8-naphthalimide transfer to a more hydrophobic environment [41]. The emission spectra of membrane-bound NAPHT-BP100 in high or low salt were similar (Figure 2B), suggesting that the fluorophore was inside the membrane.

The free/bound ratios of membrane association of NAPHT-BP100 were calculated from the lipid concentration-dependence of the fluorescence emission, assuming the equilibrium between solution-free and membrane-bound forms of the peptide (Figure 2C–E).

The addition of POPC LUV to NAPHT-BP100 in either low or high salt, reaching lipid/peptide ratios up to 240, also triggered a decrease in emission intensity, demonstrating the peptide–membrane interaction (Figure S6). However, only at high salt full binding was observed.

Interaction between NAPHT-BP100 and LUV containing 30–70 mol% of POPG occurs primarily due to electrostatic interactions between the negatively charged POPG and the positively charged lysine side chains of the peptide. The addition of LUV to NAPHT-BP100 decreased fluorescence emission to a minimum where further lipid additions ceased to produce spectral change, indicating that the peptide was fully liposome-bound.

From binding isotherm (Figure 2D,E), apparent affinity constants (K_App_) were calculated, as previously described (Methods Section 2.5). Increasing POPG content in LUV composition, increased NAPHT-BP100 binding; and the binding did not vary to a large extent with salt (Table 1). K_App_ was slightly less dependent on the POPG content at high salt. Note the differences between peptide affinity to LUV with 30 and 50 mol% of POPG (Table 1). Increasing the temperature from 25 to 65 °C did not change the binding degree (Figure S5B).

**Figure 3:**
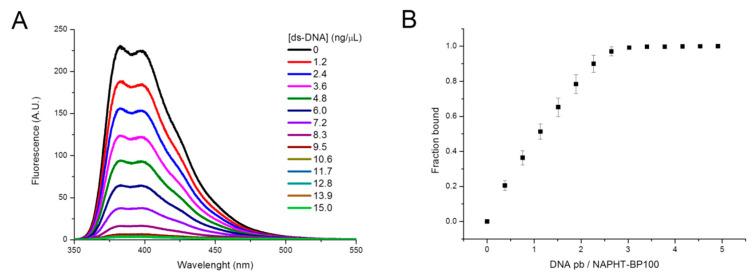
Analysis of NAPHT-BP100/ds-DNA binding. (**A**) Fluorescence spectra of NAPHT-BP100 in solution and at different concentrations of ds-DNA in Tris-HCl 10 mM, pH 7.4, buffer. (**B**) Binding curve of NAPHT-BP100 to ds-DNA in 10 mM Tris-HCl buffer, pH 7.4. The peptide concentration was 5 µM and temperature 25 °C.

Naphthalimide derivative fluorescence spectra are also sensitive to the interaction of these molecules with mono- and oligonucleotides [42]. Taking advantage of this property, NAPHT-BP100 binding to ds-DNA was investigated by fluorescence spectroscopy (Figure 3). The presence of ds-DNA in solution triggered a decrease in naphthalimide fluorescence emission attesting the peptide–DNA interaction (Figure 3A), and the degree of binding could be further explored by varying the ds-DNA concentration to obtain a binding isotherm in the same way as accomplished in the studies with LUV (Figure 3B).

NAPHT-BP100 bound to a large extent to ds-DNA, considering that complete binding was achieved at a ds-DNA base pair/NAPHT-BP100 ratio of 2.5. At this ratio, NAPHT-BP100 five positively charged groups were stoichiometrically neutralized by five negatively charged phosphate groups of the 2.5 ds-DNA base pairs. In this case, both charge neutralization and naphthalimide intercalation in the DNA [27] would contribute to a large degree of interaction.

#### 3.1.2. Circular Dichroism—Secondary Structure Analysis

**Figure 4:**
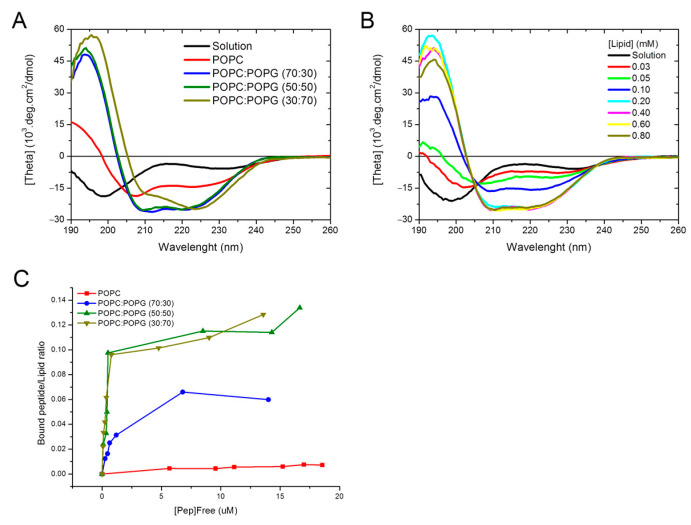
(**A**) CD spectra of NAPHT-BP100 in solution and in the presence of 0.6 mM of LUVs composed of POPC and POPG at different proportions, in 10 mM Tris-HCl buffer, pH 7.4. (**B**) CD spectra of NAPHT-BP100 in solution with different concentrations of POPC:POPG (50:50, molar ratio) in 10 mM Tris-HCl buffer, pH 7.4. (**C**) Binding isotherms of NAPHT-BP100 to LUV in 10 mM Tris-HCl buffer, pH 7.4. The peptide concentration was 20 µM and temperature 25 °C.

NAPHT-BP100 in aqueous solution displayed a far-UV CD spectrum with a negative peak at 198 nm and a negative and low-intensity band centered around 230 nm, indicating that the peptide was essentially in a random/flexible structure with a low degree of secondary structure (Figure 4A). This conformation was different from that of BP100 that, under the same conditions, displays a completely flexible structure [15,17]. Naphthalimide addition to the N-terminus of BP100 in NAPHT-BP100 induced the acquisition of some degree of secondary structure.

The CD spectra of NAPHT-BP100 bound to LUV composed of 30, 50, or 70 mol% POPG, display a positive peak at 195 nm and two negative bands at 208 and 222 nm, characteristic of a α-helix secondary structure (Figure 4A). The interaction of NAPHT-BP100 and other related peptides [13,14,15,16,17] with negatively charged vesicles triggered a coil-to-helix transition. The 208/222 nm intensity peak ratio of the spectra of NAPHT-BP100 with 70 mol% of POPG LUV differed from the ratios calculated from the spectra of the peptide bound to LUV containing 30 or 50 mol% of POPG. These differences suggested that NAPHT-BP100 might aggregate on the membrane surface and induce lipid clustering.

With LUV’s composed of POPC, NAPHT-BP100 CD spectrum changes indicated a coil-to-helix transition (Figure 4A), but complete binding was not obtained even at a lipid/peptide ratio of 190. Previous SS-NMR results with BP100 showed that the membrane-bound BP100 and other analogs adopt a helical secondary structure regardless of membrane surface net charge [43].

The isodichroic point at 204 nm indicated that the NAPHT-BP100-POPG:POPC (50:50, molar ratio) LUV interaction was compatible with a two-state model for binding (Figure 4B).

Peptide binding extent could also be examined by CD (Figure 4C, Table 1). CD data are in agreement with steady-state fluorescence studies indicating that peptide-membrane affinity increased with POPG content (Table 1).

Deconvolution of CD spectra allowed quantitative analysis of the secondary structure of NAPHT-BP100 (Table 2) [44]. In solution, NAPHT-BP100 displayed 81% of the flexible random coil structure (10.5 residues) and 19% of α-helical secondary structure (2.5 residues). Binding to lipid membranes containing POPG triggered a structural transition in which NAPHT-BP100 acquires approximately 75–83% of helical conformation (10 residues), and 17–25% of its length remaining in a random coil structure. Interestingly, deconvolution of CD spectrum of the of NAPHT-BP100 associated to LUV containing 70% of POPG suggests the peptide aggregation and the presence of more than one bound form of the peptide with different secondary structures. Aggregation could occur through the formation of β-sheet structured segments. The NAPHT-BP100 spectrum in the presence of POPC LUV was not analyzed since complete peptide binding was not achieved, and thus no representative spectrum of the peptide bound form was obtained. 

#### 3.1.3. Isothermal Titration Calorimetry—Interaction Thermodynamics

**Figure 5:**
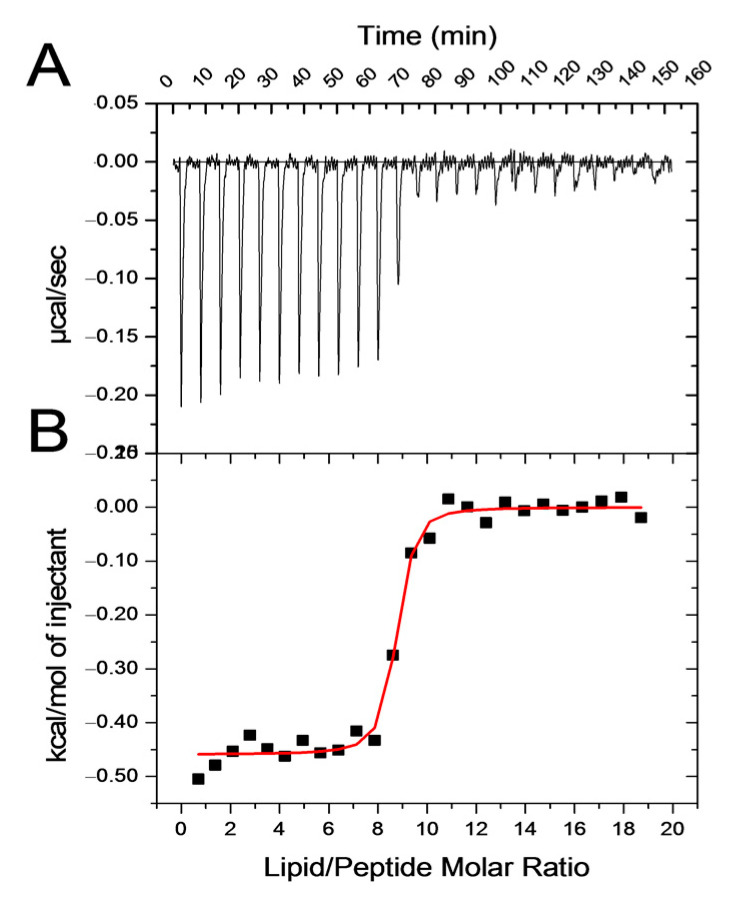
NAPHT-BP100 lipid binding by calorimetry. (**A**) Heat variation per second during titration of an NAPHT-BP100 peptide solution with a POPC:POPG (50:50) LUV suspension. (**B**) Integrated heat per mole of lipid at each injection as a function of lipid/peptide molar ratio. Measurements were performed at 25 °C, loading a 3–6 mM LUV suspension in the syringe and titrating a 40 µM peptide solution in 10 mM Tris-HCl buffer, pH 7.4.

NAPHT-BP100 binding to LUV was exothermic (Figure 5). Measured heat changes allowed for an analysis of the binding thermodynamics (Table 3).

The calorimetric data indicated that NAPHT-BP100 binding was driven by a negative enthalpy component and a positive entropy contribution (Table 3). Enthalpy variation can be ascribed to electrostatic interactions between NAPHT-BP100 and the membrane surface, while entropy variations are most likely related to peptide dehydration and hydrophobic interactions between peptide hydrophobic helix face and lipid acyl chains. Binding of some hydrotropic ions to zwitterionic interfaces is controlled by the dehydration of their hydrophobic moieties [45].

Lipid/peptide ratios where NAPHT-BP100 is fully bound are related to the stoichiometry of positively charged lysine side chains in the peptide and POPG. At a lipid/peptide ratio of ca. 10, the POPG/peptide ratio was five with LUV’s containing equal amounts of POPC:POPG (Table 3). Under these conditions, the ratio between the number of negative charges in POPG and positive charges in the peptide is 1, and the system is electroneutral. With (70:30) POPC:POPG complete peptide binding occurs at a lipid/peptide ratio of ca. 21, a condition for electroneutrality.

### 3.2. NAPHT-BP100 Effect Over the Membrane—Action Mechanism

#### 3.2.1. Carboxyfluorescein Leakage—Peptide Permeabilizing Activity

As BP100 and its analogues, NAPHT-BP100 causes CF leakage from lipid vesicles. Results are expressed in terms of lipid/peptide ratios because, as previously demonstrated [46], the extent of vesicle permeabilization depends on the peptide/lipid ratio.

**Figure 6:**
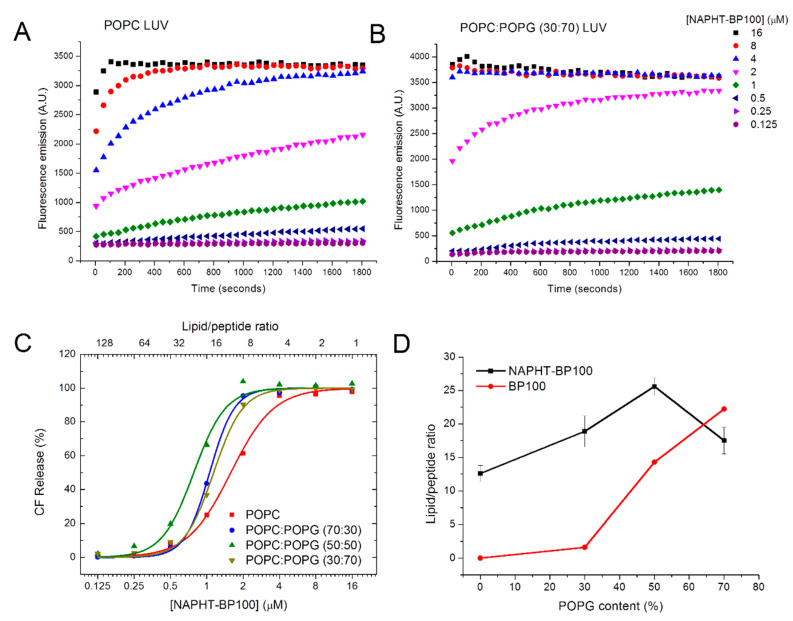
CF-induced leakage by NAPHT-BP100. Lipid concentration was 20 µM in Tris-HCl 10 mM, pH 7.4, with 300 mM NaCl. (**A**) POPC LUV and (**B**) POPC:POPG (30:70) LUV. (**C**) Percentage of CF release from LUV of varied lipid composition as a function of Napht-BP100, after 30 min. (**D**) The lipid/peptide ratio required to permeabilize 50% of LUV as a function of POPG content. Measurements performed at 37 °C.

NAPHT-BP100-induced membrane permeability increased with peptide concentration (Figure 6A,B). At a lipid/peptide ratio of 10, which, according to binding studies (Section 3.1, Figure 2D), all peptides were bound to POPC:POPG (50:50, molar ratio) LUV, the peptide caused 100% CF leakage (Figure 6C).

NAPHT-BP100 permeabilized POPC LUV, but the incorporation of POPG in LUV composition determined a greater leakage extent. Permeabilization extent and efficiency were analyzed by calculating the lipid/peptide ratio that caused 50% CF leakage (L/P_50_). The calculated L/P_50_ ratios increased with POPG (Figure 6D). With a lipid/peptide ratio of 20, where NAPHT-BP100 is bound to LUV, the leakage extent increased from 24.9 to 43.6 and 66.4% by increasing the POPG content from 0 to 30 and 50 mol%. Lower permeabilization of POPC LUV confirmed both CD and fluorescence binding results, which showed that NAPHT-BP100 interacts with a zwitterionic bilayer composed of POPC, although to a much lower extent when compared to POPG-containing membranes.

Comparing BP100 with NAPHT-BP100, it is clear that the additional alanine residues and naphthalimide increased the permeation effectiveness of NAPHT-BP100 and decreased the dependence of POPG content for binding and permeabilization.

#### 3.2.2. Dynamic Light Scattering—Peptide Effect over Liposome’s Size and Surface Charge

**Figure 7:**
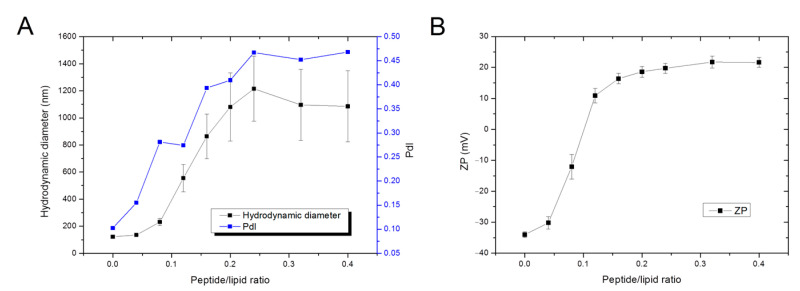
Effect of NAPHT-BP100 over LUV’s hydrodynamic diameter (Dh) and Zeta potential (ZP). (**A**) Dh and polydispersity (PdI), and (**B**) ZP of POPC:POPG (50:50) LUV, as a function of peptide/lipid ratio. Lipid concentration was 50 µM in 10 mM Tris-HF buffer, pH 7.4. Temperature was 25 °C.

NAPHT-BP100 triggered vesicle aggregation at peptide/lipid ratios above 0.12, and the apparent hydrodynamic diameter (Dh) increased to 1200 nm. The Dh increase was accompanied by an increase in the size distribution, as shown by the values of PdI and the measurement error (Figure 7).

Vesicle aggregation was correlated to the membrane net surface charge by analyzing Zeta potential (ZP) variations as the peptide/lipid ratio is increased (Figure 7B). Vesicle aggregation is directly related to membrane charge neutralization caused by positively charged peptide binding to the negatively charged membrane. At a 0.12 peptide/lipid ratio, the membrane surface charge is neutralized, and, without vesicle-to-vesicle charge repulsion, LUV aggregated. Membrane surface charge varied from −35 to +20 mV when the peptide/lipid ratios changed between 0.10 and 0.15; beyond this point, added NAPHT-BP100 did not interact with vesicle aggregates, probably due to electrostatic repulsion, and no further Dh or ZP changes were observed (Figure 7B).

#### 3.2.3. Differential Scanning Calorimetry—Peptide Effect Over Lipid Packing

**Figure 8:**
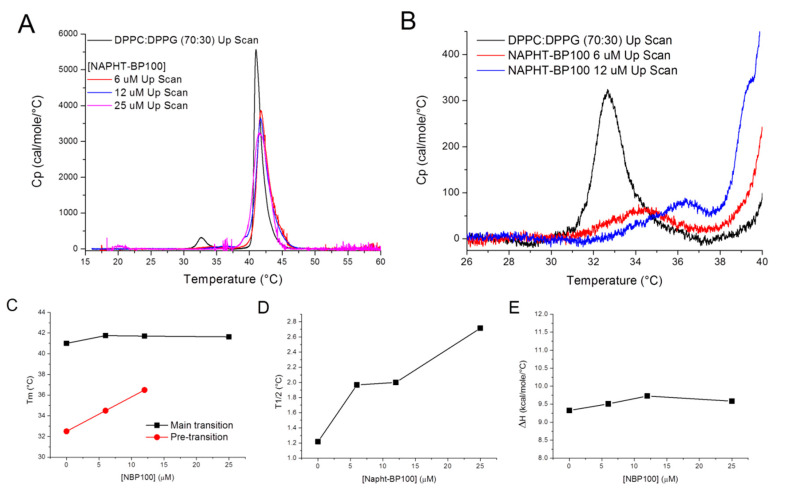
NAPHT-BP100 effects on lipid organization. Lipid concentration was 1.0 mM in 10 mM Tris-HCl buffer, pH 7.4. (**A**) Differential scanning calorimetry heating scans of DPPC:DPPG (70:30, mol:mol) multilamellar vesicle (MLV) in solution and with of various NAPHT-BP100 concentrations. (**B**) Expanded scale heating scans. (**C**) Main and prephase transition temperatures, (**D**) ΔT_1/2_ of main phase transition peak, and (**E**) phase transition enthalpy of MLV as a function of NAPHT-BP100 peptide concentration.

MLV composed of mixtures of DPPC and DPPG exhibited a pretransition (32.5 °C) from an Lβ′- to a Pβ′-phase (both gel phases), and the main transition at 40.9 °C to the liquid-crystalline Lα-phase (Figure 8). At this pH, DPPC and DPPG mixtures behaved nearly ideally in both phases and, as evidenced by the narrow main transition, indicate a process of high cooperativity [47,48].

Prephase transition was upshifted with 25 µM NAPHT-BP100 (lipid/peptide ratio 80) and superimposed with the main phase transition (Figure 8A,B,C). The addition of 6, 12, or 25 µM NAPHT-BP100 upshifted the main phase transition by 0.7 °C (Figure 8A,C), decreased the cooperativity and increased the value of ΔT_1/2_ (Figure 8A,D). The phase transition enthalpy of DPPC:DPPG mixed vesicles at pH 7.4 was 9 kcal/mol and peptide addition did not significantly alter enthalpy (Figure 8E). The limited destabilization of the DPPC:DPPG gel phase by NAPHT-BP100 may be related to its interfacial position in the bilayer [8].

### 3.3. Biological Activity

#### 3.3.1. Minimum Inhibitory Concentration

The additional two alanine residues and the naphthalimide group linked to the BP100 sequence improved the peptide’s ability of inhibit bacterial growth against *E. coli* and *S. aureus* and did not alter the activity against *B. subtilis* (Table 4). No exact correlation regarding improving activity and species Gram stain could be drawn. The higher inhibitory activity of NAPHT-BP100, in comparison with BP100, correlates with NAPHT-BP100 greater membrane affinity and could relate to NAPHT-BP100 ability to bind bacterial cytoplasmic DNA.

#### 3.3.2. Hemolytic Activity

**Figure 9:**
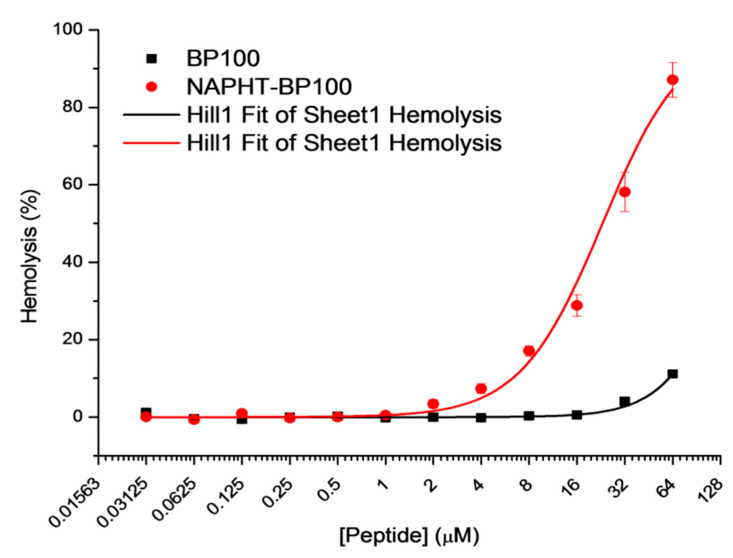
Hemolytic activity of BP100 (black) and NAPHT-BP100 (red) as function of peptide concentration. Red blood cells 1.5% (*v*/*v*) in PBS, pH 7.0. Temperature was 37 °C.

NAPHT-BP100 caused significant hemolysis above 8 µM (Figure 9). Compared to BP100 [17] (Fit projected C_50_ = 163 µM), NAPHT-BP100 displayed greater toxicity (C_50_ = 23 µM). This effect correlates with the attested higher membrane binding extent of NAPHT-BP100. Studies with the POPC zwitterionic model membrane, an adequate lipid membrane composition to correlate with neutral RBC cell membrane, showed that NAPHT-BP100 binds to and permeabilizes LUV to a greater extent than BP100.

## 4. Discussion

Synthesis, properties, and biological activities of amphiphilic peptides covalently linked to groups of diverse chemical structures are of fundamental and applied interest [3,17,49,50].

Membrane-NAPHT-BP100 binding was extensively studied using different techniques (Figure 2, Figure 4 and Figure 5 and Table 1 and Table 3). Altogether, our results allowed the quantification of the binding extent and the measurement and description of the thermodynamic parameters of the interaction. Fluorescence emission spectrum of the naphthalimidic group of the peptide varied both quantitatively and qualitatively as the LUV concentration increased (Figure 2). Non-substituted 1,8-naphthalimide presents higher quantum yields in polar protic solvents when compared to a hydrophobic aprotic environment [38]; thus, changes in emission can be attributed to the fluorophore passage from the bulk solution to the more hydrophobic and less hydrated membrane surface environment. A significant blue-shift of the spectra was also observed as NAPHT-BP100 bound to LUV and, likely, accommodated at the hydrophobic core of the bilayer. Upon interaction with ds-DNA, NAPHT-BP100 fluorescence emission also decreased but no blue-shift was observed (Figure 3); such variation can be interpreted as an indication that NAPHT-BP100 bound and intercalated to ds-DNA experiencing a less hydrated, thus the lower emission, but considerably polar environment the ds-DNA. CD spectra of the peptide at different lipid concentrations also varied quantitatively and qualitatively (Figure 4), and, in this case, changes are attributed to conformational changes occurring as NAPHT-BP100 passes from the bulk solution to the membrane. CD and fluorescence binding data were in good agreement and clearly indicate that NAPHT-BP100 was able to bind to POPC LUV to a low extent and the binding extent increased in a POPG content-dependent manner, highlighting the importance of electrostatic interactions between the positively charged lysine residue side chains and negatively charged phosphate groups of POPG. In comparison with BP100, NAPHT-BP100 presented higher affinity to LUV and reduced charge dependence. The extra two alanine residues used as a spacer and the aromatic rings of the naphthalimide moiety at the BP100 N-terminal increased molecular hydrophobicity and contributed to the peptide–membrane interaction by decreasing the energy to accommodate the peptide on the bilayer hydrophilic/hydrophobic interface regardless of the membrane surface charge. NAPHT-BP100, differently from BP100, was able to bind to POPC LUV. Helix stabilization given by the addition of the alanine residues at the BP100 N-terminal sequence would also play an important role in increasing peptide–membrane interactions.

ITC data analysis confirmed that the peptide–membrane interaction is essentially driven by electrostatic interactions (Figure 5, Table 3), as shown by CD and fluorescence results. Measured negative enthalpy variation is mostly associated with charge neutralization. The role of electrostatic interactions was made even clearer as the calculated stoichiometry of the interaction is essentially given by one molecule of POPG to one lysine side chain of the peptide, as demonstrated (Table 3).

The main phenomena associated with entropy variation are peptide dehydration and secondary structure acquisition and the hydrophobic effect driving the interaction between the α-helix non-polar face and lipid acyl chains. Although helix acquisition represents a configurational entropy loss, peptide dehydration, and hydrophobic effects increase the overall entropy, resulting in a significant energetic contribution to the total free energy variation.

The secondary structure of NAPHT-BP100 in water and bound to LUV was investigated by CD (Figure 4, Table 2). CD spectra showed that the peptide underwent a coil to α-helix transition upon binding to LUV containing POPG.

In solution, NAPHT-BP100 presents a small content of α-helix (19%, or 2.5 residues) and is essentially in a flexible random conformation (81%, or 10.5 residues) (Figure 4, Table 2). The small but significant content of the secondary structure is most likely an effect of the Napht-Ala-Ala segment attached to the BP100 sequence considering that, in solution, BP100 is completely in random conformation [8,14,15,16,51]. Addition of chemical groups to the BP100 N-terminal affected its structure in water by enhancing the content of α-helix [17] and alanine residues are known to play a role as α-helix stabilizers [52].

**Figure 10:**
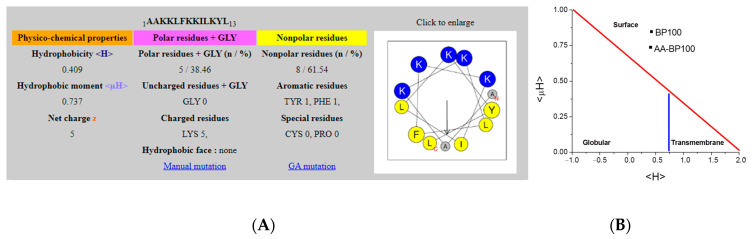
(**A**) NAPHT-BP100 peptide sequence and formed α-helix properties calculated using Heliquest [54]. (**B**) BP100 and NAPHT-BP100 α-helices properties placed in the Eisenberg plot.

Binding to lipid bilayers containing POPG triggered the acquisition of a α-helical structure (80%, or 10.4 residues) (Figure 4, Table 2). Conformational changes are directly related to peptide dehydration occurring as peptides interact more closely with the bilayer surface, and to the lower availability of water molecules at the membrane surface that trigger the establishment of intermolecular hydrogen bonds in the peptide that adopts a α-helical secondary structure [8,53]. Helical wheel projection and theoretical calculations of NAPHT-BP100 indicate that the peptide, BP100, forms an amphipathic α-helix with two extra alanine residues attached to the N-terminal end is positioned in the hydrophobic face of the structure [54] (Figure 10). According to the Eisenberg plot [55], NAPHT-BP100 overall hydrophobicity (<H> = 0.409) and significant hydrophobic moment (<µH> = 0.737) classify the peptide-formed helix as “membrane surface seeking”. In addition, it has been reported that slight variations in peptide sequence, thus in its hydrophobicity and hydrophobic moment, can trigger considerable changes in the peptide–membrane interaction and permeabilizing capabilities of the peptide [56,57].

Changes in liposome size and size distribution, and zeta potential triggered by NAPHT-BP100 were measured using dynamic light scattering (Figure 7), revealing that membrane charge neutralization plays an important role in membrane destabilization, ultimately causing lipid aggregation. The relationship between liposome charge neutralization and interaction stoichiometry could be detailed by zeta potential measurements, indicating that most of the peptide effect occurs in the lipid to peptide ratio range in which the Zeta potential is close to 0 mV and the sample has approximately one molecule of POPG to each lysine side chain of the peptide. This observation corroborates the stoichiometry calculated from ITC data (Table 3) and brings relevant information regarding NAPHT-BP100 action mechanism, suggesting a detergent-like mechanism in which charge neutralization triggers LUV aggregation and complete disruption.

The analysis of the secondary structure amphipathic profile indicated that the peptide tends to occupy an interfacial position in the bilayer. DSC experiments showed that the peptide does not cause a major effect on gel phase lipid organization, supporting the proposed shallow penetration (Figure 8). Thermodynamic lipid phase transition parameters of DPPC:DPPG (70:30) MLV and the effect of the peptide on these parameters were examined by differential scanning calorimetry (Figure 8). Essentially, no significant influence was observed, especially regarding the overall process energy and the main transition temperature.

CF leakage assays to test peptide efficiency for permeabilizing LUV were evaluated considering the binding extent and structure of the peptide in the bilayer, the bilayer surface charge neutralization, and the shallow penetration of the peptide on lipid acyl chains (Figure 6). Peptide activity efficiency followed the previously measured binding extent in terms of both the lipid/peptide ratio and LUV POPG content. Although low binding of NAPHT-BP100 to POPC LUV was demonstrated, the peptide was able to bind and cause CF leakage from these liposomes, confirming the presence and relevance of hydrophobic interactions and other phenomena not associated with electrostatic interactions.

Measured biological activity of NAPHT-BP100 against bacteria and human RBC corroborates the series of biophysical studies discussed so far. Experiments with LUV composed of 30 mol% of POPG, generally mimicking PG molar concentrations found in bacteria membrane, correlated with the MIC results. Observations taken in experiments with zwitterionic POPC LUV were correlated to the biological hemolytic activity assay considering the human RBC have neutral membrane surface. In comparison with BP100, NAPHT-BP100 showed higher affinity to all studied LUV compositions, resulting in a higher effect on the membrane regardless of its composition. These observations can be translated to the observed greater biological activity of NAPHT-BP100 against *E. coli* and *S. aureus*, confirmed by the measured lower MIC values (Table 4), and to greater toxicity against human RBC as demonstrated by the hemolysis test (Figure 9). Increased activity of NAPHT-BP100 against *E. coli* and *S. aureus* could also relate to the internalization of NAPHT-BP100 into the bacteria and NAPHT-BP100-ds-DNA interaction, resulting in DNA replication and RNA transcription blockage. NAPHT-BP100 bound to a large extent to ds-DNA (Figure 3) and naphthalimide derivatives are known to be able to intercalate between ds-DNA base pairs that affect a series of related cellular processes [27].

The increase in hemolytic activity of NAPHT-BP100, compared to BP100, was balanced by its improved antibacterial action and did not change to a large extent the peptide’s therapeutic index. The indication that NAPHT-BP100 could act upon bacteria not only by destabilizing its membrane but also by binding the cell ds-DNA, means that the peptide could act by more than a single mechanism, would also consist in a considerable improvement in terms of avoiding bacterial resistance. Although more hemolytic, NAPHT-BP100 can be applied to the design of antibacterial molecules targeting various types of cutaneous or other mucosal infections. In this context, the results found in the present work can provide subsidies for studies aimed at drug development.

## Figures and Tables

**Table 1:** Apparent binding constants (K_App_, ×10^5^ M^−1^) calculated fluorescence and circular dichroism (CD) data.

	Method	Fluorescence		CD
LUV POPG Content (%)		No Salt	0.3 M NaCl	No Salt
0		0.01	0.05	0.005
30		0.8	0.7	0.5
50		2.4	2.0	1.5
70		5.1	5.2	2.8

**Table 2:** Secondary structure content calculated from CD spectra of NAPHT-BP100 in solution and bound to LUV.

Lipid.	α-Helix (%)	β-Sheet (%)	Flexible (%)
None	19	0	81
POPC:POPG 70:30	76	6	18
POPC:POPG 50:50	83	0	17
POPC:POPG 30:70	75	17	8

**Table 3:** Thermodynamic parameters of NAPHT-BP100-lipid interaction.

PG%	N (Lip/NAPHT-BP100)	ΔH (kcal/mol)	ΔS (cal/mol/K)	−T ΔS (kcal/mol)	ΔG (kcal/mol)	K (M^−1^)
30	21.7	−5.4 ± 0.3	9.8 ± 2.5	−2.9	−8.3	1.4 ± 1.0 × 10^6^
50	10.6	−5.1 ± 1.3	14.1 ± 4.8	−4.2	−9.3	3.2 ± 0.6 × 10^7^

**Table 4:** Minimum inhibition concentration (MIC) values for BP100 and NAPHT-BP100.

	BP100	NAPHT-BP100
*E. coli*	2 µM	1 µM
*S. aureus*	2 µM	1 µM
*B. subtilis*	2 µM	2 µM

## Data Availability

Not applicable.

## Supplementary Materials

The following are available online at https://www.mdpi.com/article/10.3390/biom11040542/s1, Figure S1: Analytical chromatographic profile of freeze dried crude Naphthalimide-AA-BP100, Figure S2: Analytical chromatographic profile of purified Naphthalimide-AA-BP100, Figure S3: MALDI mass spectra of the purified peptide Naphthalimide-AA-BP100, Figure S4: MS/MS spectrum of the peptide Naphthalimide-AA-BP100, Figure S5: (A) Fluorescence spectra of NAPHT-BP100 20 µM in solution at 25, 45 and 65 °C in 10 mM Tris-HCl buffer, pH 7.4. (B) Average lipid/peptide ratio in which 50% of NAPHT-BP100 is bound to LUV of varied lipid composition at 25, 45, and 65 °C, Figure S6: (A) Fluorescence and (C) CD spectra of NAPHT-BP100 20 µM in solution and in presence of POPC LUV, in 10 mM Tris-HCl buffer, pH 7.4. (B) Binding isotherm obtained from the spectra presented in (A) and (D) [θ] at 222 nm as function of lipid concentration obtained from data in (C).
