# Supplementary material for: Naphthalimide-Containing BP100 Leads to Higher Model Membranes Interactions and Antimicrobial Activity
Source: Biomolecules. 2021 Apr 8;11(4):542. doi: 10.3390/biom11040542 (PMC8068292; doi:10.3390/biom11040542)
Supplement: Supplementary file 1 [file biomolecules-11-00542-s001.pdf]

Supplementary material

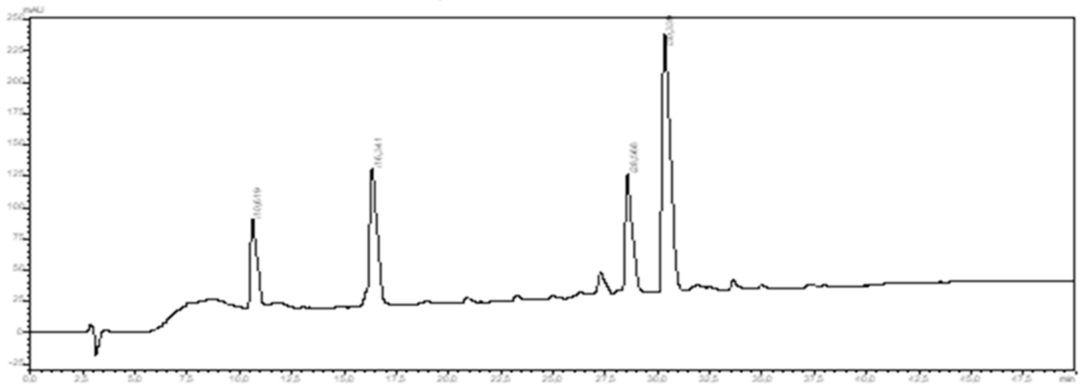

Figure SM-1. Analytical chromatographic profile of freeze dried crude Naphthalimide-AA-BP100 - 2 mg/mL in water; sample volume: 20 µL; analytical reversed-phase C18 Vydac column; solvent A: 0.1% TFA in water; solvent B: 80% aqueous acetonitrile containing 0.09 % TFA; gradient: 30-70 % B in 30 min; flow rate: 1 mL/min: detection wavelength: 220 nm. This peptide was constituted by four fractions which were separated from each other and purified separately using a preparative reversed-phase C18 Vydac column at flow rate: 9 mL/min; in the same experimental conditions of solvent and gradient above. Mass spectra analyses of each fractions indicated that peak 4 was the desired peptide ([M+H]+ = 1743.1).

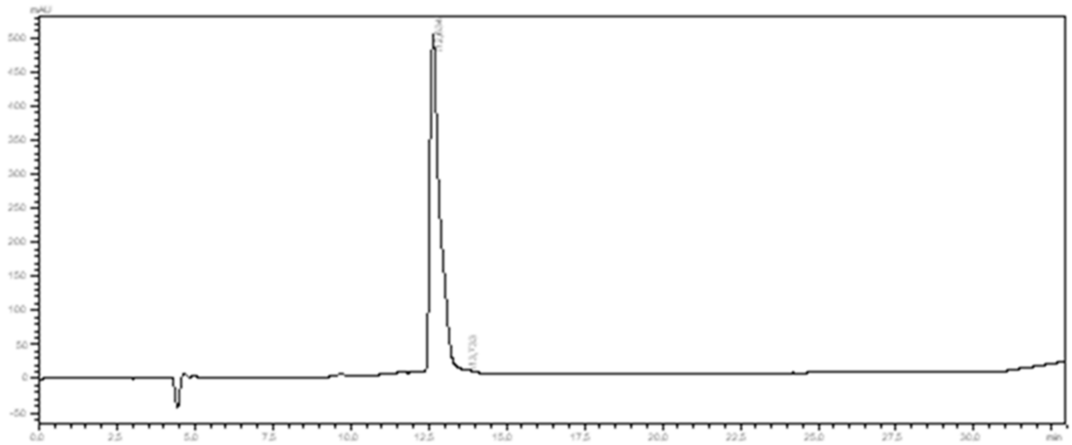

Figure SM-2. Analytical chromatographic profile of purified Naphthalimide-AA-BP100 (peak 4)- 2 mg/mL in water; sample volume: 20 uL, analytical reversed-phase C18 Vydac column; solvent A: 0.1% TFA in water; solvent B: 80% aqueous acetonitrile containing 0.09 % TFA; gradient: 30-40%B in 5 min plus 40-50%B in 20 min; flow rate: 1 mL/min: detection wavelength: 220 nm. HPLC purity grade: >99 %.

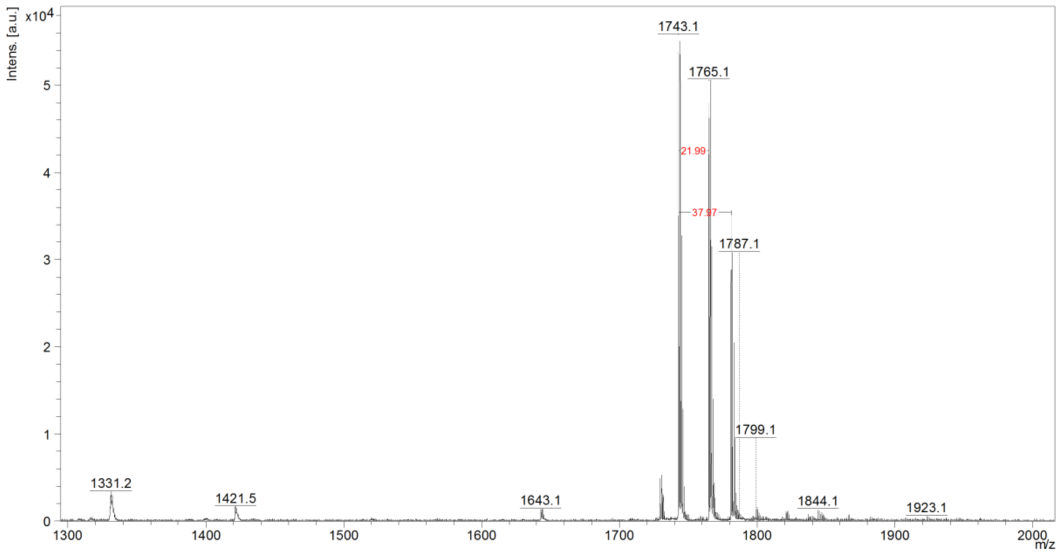

Figure SM-3. MALDI mass spectra of the purified peptide Naphthalimide-AA-BP100, showing the expected product with $[M+H]^+$= 1743.1 and the $[M+Na]^+$ and $[M+K]^+$ adducts at 1765.1 1nd 1787.1, respectively. Ions observed at *m/z* values of 1331.2, 1421.5 and 1643.1 might be due to partial in-source peptide fragmentation. The spectrum was obtained in an Autoflex Speed mass spectrometer (Bruker Daltonics, Bilerica, MA).

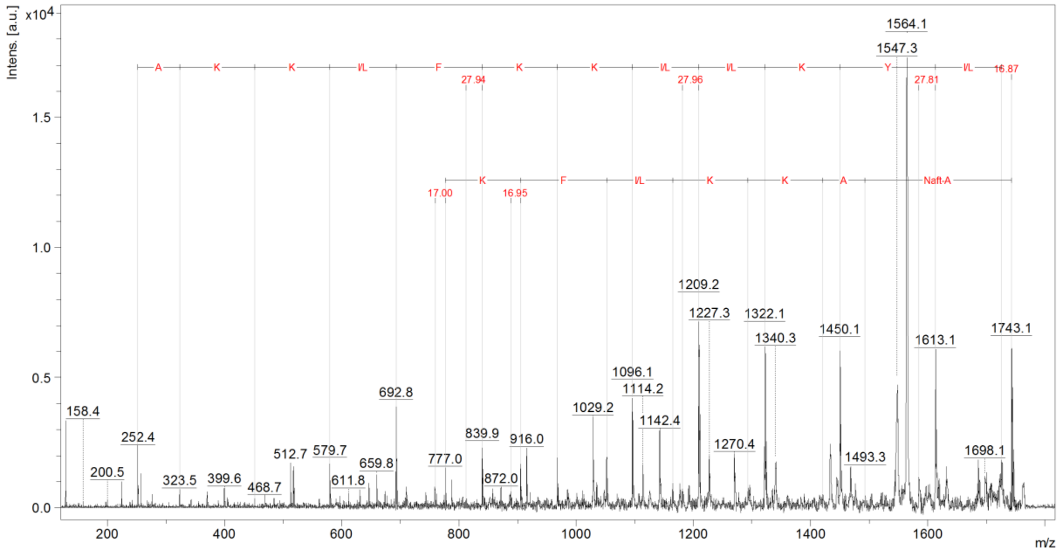

Figure SM-4. MS/MS spectrum of the peptide Naphthalimide-AA-BP100. The complete sequence obtained by the b ion-series is shown in the upper part whereas a partial sequence assigned by the y ion-series is shown below the b-series. The spectrum was obtained in an Autoflex Speed mass spectrometer (Bruker Daltonics, Bilerica, MA).

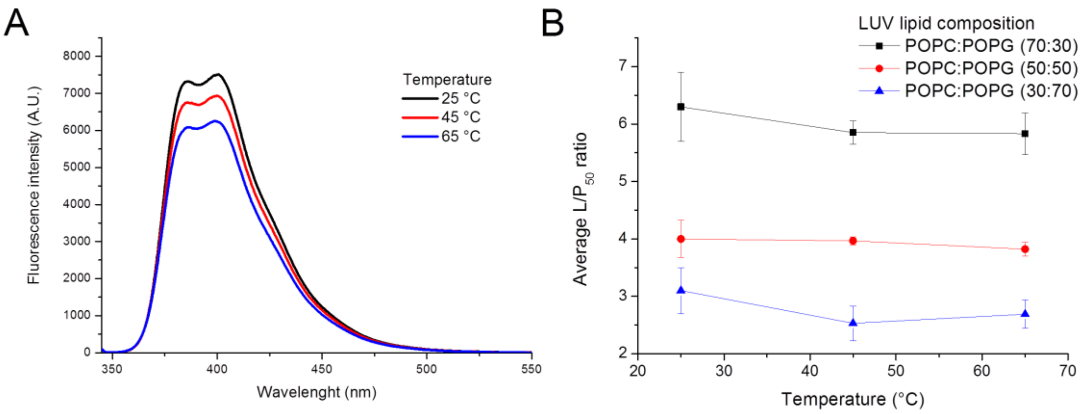

Figure SM-5. (A) Fluorescence spectra of NAPHT-BP100 20 μM in solution at 25, 45 and 65 °C in 10 mM Tris-HCl buffer, pH 7.4. **(B)** Average lipid/peptide ratio in which 50% of NAPHT-BP100 is bound to LUV of varied lipid composition at 25, 45, and 65 °C.

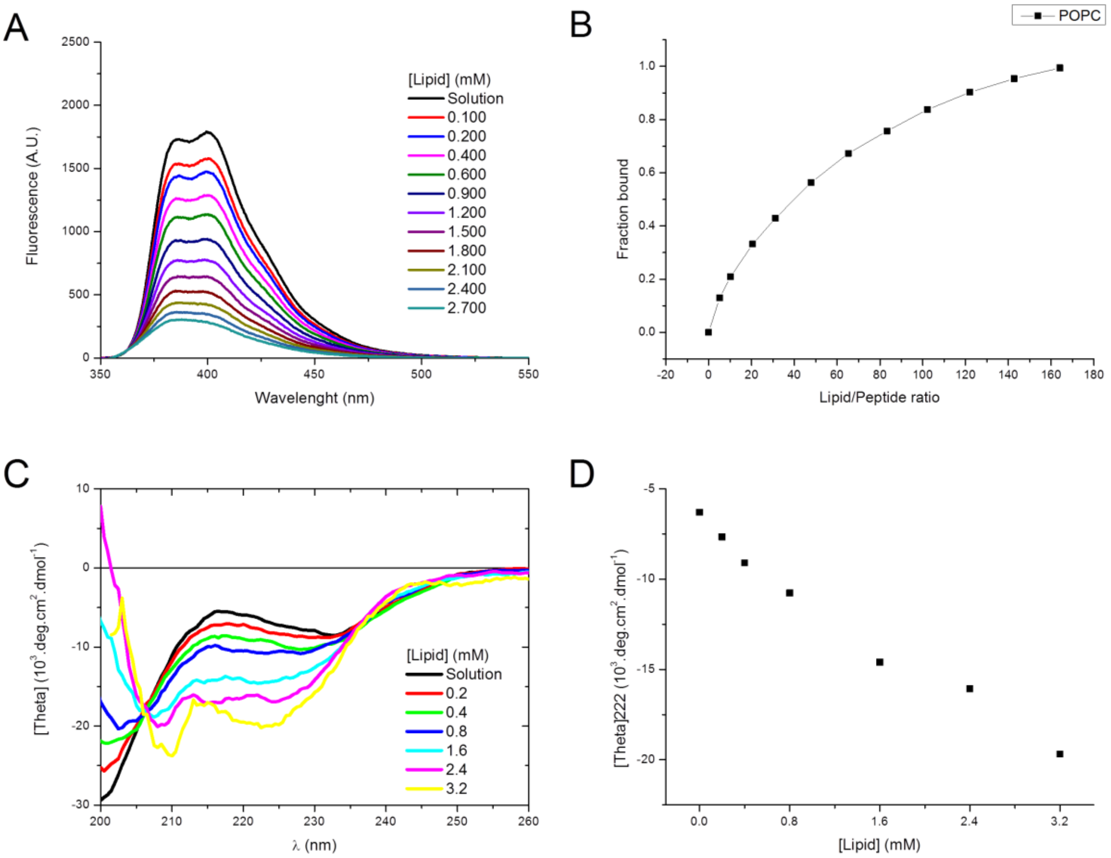

Figure SM-6. (A) Fluorescence and **(C)** CD spectra of NAPHT-BP100 20 μM in solution and in presence of POPC LUV, in 10 mM Tris-HCl buffer, pH 7.4. **(B)** Binding isotherm obtained from the spectra presented in (A) and **(D)** [θ] at 222 nm as function of lipid concentration obtained from data in (C).

1
